# Evaluation of large airway specimens obtained by transbronchial lung cryobiopsy in diffuse parenchymal lung diseases

**DOI:** 10.1186/s12890-022-02186-6

**Published:** 2022-10-18

**Authors:** S. Sato, H. Yamakawa, T. Takemura, T. Nakamura, T. Nishizawa, T. Oba, R. Kawabe, K. Akasaka, M. Amano, H. Matsushima

**Affiliations:** 1 Department of Respiratory Medicine, Saitama Red Cross Hospital, 1-5 Shintoshin, Chuo-ku, Saitama, 330-8553 Japan; 2 Department of Pathology, Kanagawa Cardiovascular and Respiratory Center, Kanagawa, Japan

**Keywords:** Large airway specimen, Histological diagnostic yield, Specimen adequacy, Surgical lung biopsy, Transbronchial lung cryobiopsy

## Abstract

**Background:**

The difference in diagnostic yield between surgical lung biopsy and transbronchial lung cryobiopsy (TBLC) in diffuse parenchymal lung diseases (DPLD) has been reported to be due to differences in the rate of interpathologist agreement, specimen size, and specimen adequacy. In TBLC, the specimens containing large airway components are generally believed as inadequate specimens for histological evaluation, but the detailed characteristics of TBLC specimens including the large airway and the impact on histological diagnostic rates of DPLD have not been investigated.

**Methods:**

We retrospectively reviewed the specimen characteristics of patients with DPLD who underwent TBLC.

**Results:**

Between February 2018 and January 2020, 74 patients and 177 specimens were included. There were 85 (48.0%) large airway specimens (LAS) that contained bronchial gland or bronchial cartilage. The ideal specimen ratio was significantly lower in the LAS-positive group than that in the LAS-negative group (5.8% vs. 45.6%), and the proportion of bronchioles, alveoli, and perilobular area were similarly lower in the LAS-positive group. The presence of traction bronchiectasis and diaphragm overlap sign on high-resolution computed tomography (HRCT) were also significantly higher in the LAS-positive group than those in the LAS-negative group. We observed a statistically significant trend in histological diagnostic yield (40.7% in LAS positive group; 60.8% in LAS positive and negative group; 91.6% in LAS negative group) (Cochran-Armitage trend test).

**Conclusion:**

LAS is a specimen often collected in TBLC and contains a low percentage of bronchioles, alveoli, and perilobular area. Since the histological diagnostic yield tends to be higher in cases that do not contain LAS, it may be important to determine the biopsy site that reduces the frequency of LAS collection by referring to the HRCT findings in TBLC.

## Background

Diffuse parenchymal lung diseases (DPLD) comprise heterogeneous disorders with different prognostic and therapeutic implications. Many international guidelines have required confirmation of the pathological findings by surgical lung biopsy (SLB) to recognize histological patterns and provide an accurate diagnosis by obtaining a sufficient volume of specimens [1, 2]. SLB has a histological diagnostic yield of more than 90%, but it has disadvantages of certain morbidity and mortality and the presence of patients excluded from the indication due to advanced age, complications including pulmonary hypertension, and severe respiratory failure [3, 4]. Transbronchial lung cryobiopsy (TBLC) is a promising new bronchoscopic biopsy technique with lower mortality and fewer complications, but the diagnostic yield for DPLD is 70–80%, which is inferior to that of SLB [5]. This difference in diagnostic yield between SLB and TBLC has been reported to be due to the rate of interpathologist agreement, specimen size, and specimen adequacy [6–9].

As mentioned in previous TBLC reports for DPLD [10, 11], specimens containing large airway are considered unideal with little contribution to the histological diagnosis. However, the specific conditions under which these specimens are likely to be collected and their actual impact on diagnostic rates are not known. The purpose of this study was to examine the characteristics of TBLC specimens containing large airway and their impact on the rate of histological diagnosis in individual cases.

## Materials and methods

### Patients

This retrospective study was conducted in the Department of Respiratory Medicine of Saitama Red Cross Hospital between February 2018 and January 2020 and was approved from the institutional review board of Saitama Red Cross Hospital (approval number 19-C). The need for patient approval and/or informed consent was waived due to the retrospective nature of the study. The inclusion criteria were age > 20 years and patients with DPLD who underwent TBLC. Exclusion criteria were as follows: patients who underwent biopsy from a site directly visible by bronchoscopy, patients for whom biopsy was performed after confirmation by endobronchial ultrasonography (EBUS) at a peripheral site, patients who met the pulmonary function test criteria for relative contraindications to TBLC as indicated in a previous report [12]; %forced vital capacity < 50% and %diffusion capacity of the lung for carbon monoxide < 35%, and patients for whom pulmonary function test, high-resolution computed tomography (HRCT), or laboratory findings were missing within 3 months of TBLC.

### Data collection

Baseline clinical measurements were obtained within 3 months of the bronchoscopy. Each patient’s HRCT scan was reviewed in a multi-disciplinary discussion and classified as presenting an HRCT pattern of usual interstitial pneumonia (UIP), probable UIP, indeterminate for UIP, or alternative diagnosis according to the American Thoracic Society (ATS)/European Respiratory Society (ERS)/Japanese Respiratory Society (JRS)/Latin American Thoracic Association (ALAT) guideline 2018 [13]. Definitions of terms related to the HRCT findings were based on the definitions of the Fleischer Society [14].

### Transbronchial cryobiopsy procedure

Bronchoscopy was performed with a BF-1T290 flexible bronchoscope (Olympus, Tokyo, Japan). Patients were anesthetized with midazolam and fentanyl intravenously, and 2% lidocaine was added intratracheally as appropriate. The endotracheal tube (SACETT Suction Above Cuff Endotracheal Tube 8.0 mm; Smiths Medical International Ltd., Minneapolis, MN, USA) was inserted for airway control. An endobronchial balloon catheter (Fogarty catheter, E-080-4F; Edwards Life-sciences, Irvine, CA, USA) was routinely used for bronchial blockade. The flexible reusable cryoprobe, either 1.9 mm or 2.4 mm in diameter (ERBECRYO® 2 system; Erbe Elektromedizin GmbH, Tübingen, Germany) was inserted from a peripheral bronchus to just below the pleura under fluoroscopy. The probe was withdrawn for 1 cm proximally to prevent pneumothorax. After freezing the probe for 5–7 s, the bronchoscope was withdrawn along with the biopsy specimen and probe, and the endobronchial balloon catheter was inflated simultaneously. In accordance with previous reports [15], Complications were classified into four levels. “Serious” adverse events were defined as those that were life-threatening, and ‘severe’ adverse events were defined as those requiring surgical or radiological interventions or mechanical ventilation. “Moderate’ or ‘mild’ adverse events were defined according to the severity of individual types of adverse events.

### Pathological evaluation

The biopsy specimen was fixed in 10% neutral buffered formalin, embedded in paraffin, and stained with hematoxylin and eosin. Additional staining and immunohistochemistry were performed when a more accurate evaluation was required. Each TBLC specimen was evaluated by a pulmonary pathology expert and classified according to the included anatomical structures (large airway, bronchioles, alveoli, perilobular area, and pleura).

Based on the fact that the bronchi divide into membranous bronchioles of 2 mm diameter without bronchial glands or bronchial cartilage at the seventh branch [16], we defined LAS as biopsy specimens in which a bronchial gland or bronchial cartilage was present. The biopsy specimens were evaluated in terms of quality and confidence level of the pathological diagnosis. We defined an ideal specimen as a specimen that includes all of bronchioles, alveoli, and perilobular area. The confidence level was classified into three groups [17]. Level A specimens could be used to establish a definite pathological diagnosis; level B specimens could be used to suggest a probable diagnosis; and level C specimens showed only certain findings that were difficult to diagnose by themselves. When a definitive diagnosis such as hypersensitivity pneumonitis, sarcoidosis, or malignancy could not be made and the disease was considered idiopathic, the ATS/ERS/JRS/ALAT guideline 2018 was applied for histological diagnosis [13]. The clinical information, HRCT findings, and pathological diagnosis were then reviewed in a multidisciplinary discussion to make the final institutional diagnosis.

### Study design

**Fig. 1:**
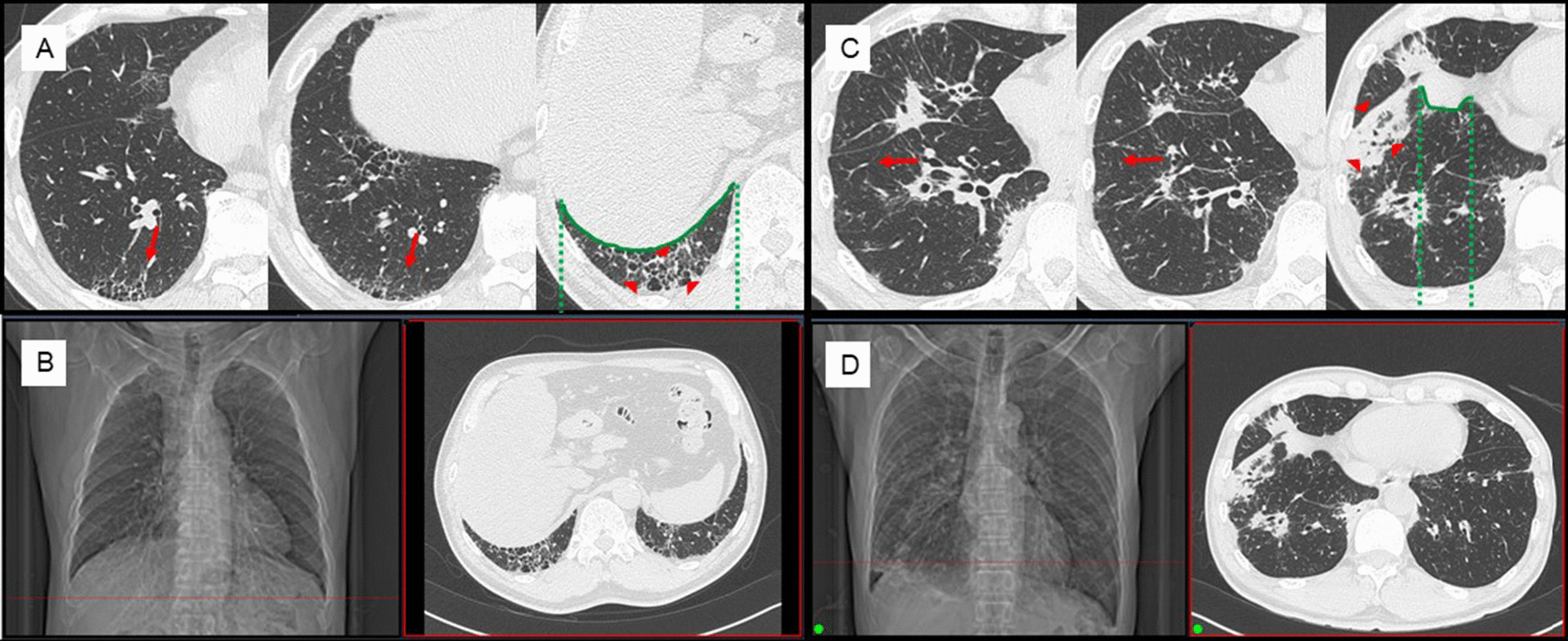
Chest HRCT findings of diaphragm overlap sign. **a** Case positive for diaphragm overlap sign. Right B^10^ b + c branched into a subsegmental bronchus (arrows); the peripheral lesion included reticular shadows and ground-glass opacities with traction bronchiectasis (arrowheads). In the anterior–posterior direction, this S^10^b + c area and the diaphragm completely overlapped (inside of the green area) and were judged to be positive for diaphragm overlap sign. **b** The S^10^b + c lesion is located at the red line in the CT scout view, and the coronal section shows that it is completely subdiaphragmatic in height. **c** Case negative for diaphragm overlap sign. Right B^8^ branched into a subsegmental bronchus, and then B^8^a proceeded laterally (arrows); the peripheral lesion of B^8^b included a consolidation (arrowheads). In the anterior–posterior direction, S^8^b did not overlap with the diaphragm (outside of the green area) and was judged to be negative for diaphragm overlap sign. **d** The S^8^b lesion is located at the red line in the CT scout view, and the coronal section shows that it to be at a height above the diaphragm

We identified the anatomical structures in all TBLC specimens and classified them into specimens with or without LAS and compared patient characteristics and specimen characteristics. Patient characteristics included sex, age, smoking history, fibrosis biomarkers, pulmonary function tests, HRCT findings, HRCT pattern, histological diagnostic yield, complications, and the number of specimens collected. Specimen characteristics included maximum diameter, biopsy lobe, structures observed in the specimen, ideal specimen ratio, pathological confidence, HRCT findings in the biopsied lung segment, and diaphragm overlap sign. In this study, we have set a new HRCT sign called diaphragm overlap sign, which examines whether the biopsy site has overlap with the diaphragm in the anterior–posterior direction on HRCT (shown in Fig. 1). When the diaphragm overlaps the lung at the biopsy site during a TBLC procedure, the position of the cryoprobe tip in the lung and its distance from the pleura are more likely to be indistinct, increasing the likelihood of obtaining a centrally sided specimen.

### Statistical analysis

Categorical variables are expressed as frequencies and percentages, and continuous variables are expressed as means and standard deviations, as appropriate. Categorical variables were analyzed using the chi-squared test, Fisher’s exact test, and Cochran-Armitage trend test. Continuous variables were analyzed using Mann–Whitney U test and one-way analysis of variance (ANOVA) with post hoc Tukey’s tests. For all statistical tests, a *P* value < 0.05 was defined as significant. Statistical analysis was performed using EZR version 1.42 (CRAN: the Comprehensive R Archive Network at http://cran.r-project.org/).

## Results

### Patients

This study included 90 consecutive patients evaluated between February 2018 and January 2020. We excluded 16 patients: 9 were missing pulmonary function tests within 3 months of TBLC, 3 were biopsied for peripheral lung lesions using EBUS, 2 were biopsied for central-type lung cancer, and 2 had an inappropriate pathological evaluation. In total, 74 patients were available in this study, and their baseline characteristics are as indicated in Table 1 for the three groups: LAS-positive, LAS-positive and LAS-negative, LAS-negative. There were 50 LAS-positive cases (67.5%), of which all specimens were LAS in 27 cases and only some specimens were LAS in 23 cases. HRCT pattern accounted for approximately 90% of indeterminate for UIP and alternative diagnoses. All cases with HRCT pattern UIP were positive for some collagen-related autoantibodies or myeloperoxidase-antineutrophil cytoplasmic antibody.

**Table 1:** Baseline characteristics of patients

Characteristics	All patients	LAS-positive	LAS-positive and LAS-negative	LAS-negative	*P* value
Patients, *n*	74	27 (36.4)	23 (31.0)	24 (32.4)	
Male, *n* (%)	40 (54.0)	14 (51.8)	15 (65.2)	11 (45.8)	0.698
Age, years	67.0 ± 11.5	69.1 ± 7.1	67.6 ± 8.6	64.0 ± 16.7	0.278
Current or ex-smokers, *n* (%)	44 (59.4)	16 (59.2)	17 (73.9)	11 (45.8)	0.358
KL-6, U/mL	1565.1 ± 1523.9	1473 ± 1051	1784 ± 2048	1455 ± 1424	0.713
SP-D, ng/mL	346.4 ± 293.0	339.7 ± 279.9	372.1 ± 339.5	327.8 ± 267.4	0.873
PFT					
%FVC	81.8 ± 20.2	78.9 ± 18.4	80.1 ± 18.0	86.8 ± 23.8	0.347
FEV_1_/FVC ratio	79.9 ± 10.2	80.4 ± 11.9	81.5 ± 8.9	77.9 ± 9.3	0.483
%DL_CO_	64.1 ± 24.5	68.0 ± 27.2	63.9 ± 23.1	60.3 ± 23.3	0.584
HRCT findings, *n* (%)					
Honeycomb	19 (25.6)	7 (25.9)	5 (18.5)	7 (29.1)	0.805
Traction bronchiectasis	50 (67.5)	22 (81.4)	15 (65.2)	13 (54.1)	**< 0.05**
Reticulation	63 (85.1)	25 (92.5)	20 (86.9)	18 (75.0)	0.07
GGO	55 (74.3)	19 (70.3)	16 (69.5)	20 (83.3)	0.3
Consolidation	17 (22.9)	5 (18.5)	6 (26.0)	6 (25.0)	0.573
Nodule	17 (22.9)	7 (25.9)	4 (17.3)	6 (25.0)	0.917
Cyst	12 (16.2)	4 (14.8)	4 (17.3)	4 (16.6)	0.853
HRCT pattern, *n* (%)					
UIP	5 (6.7)	1 (3.7)	3 (13.0)	1 (4.1)	0.91
Probable UIP	4 (5.4)	2 (7.4)	1 (4.3)	1 (4.1)	0.603
Indeterminate for UIP	27 (36.4)	14 (51.8)	7 (30.4)	6 (25.0)	**< 0.05**
Alternative diagnosis	38 (51.3)	10 (37.0)	12 (52.1)	16 (66.6)	**< 0.05**
Histological diagnosis, n (%)	47 (63.5)	11 (40.7)	14 (60.8)	22 (91.6)	**< 0.05**
Number of specimens, *n*	2.3 ± 0.7	2.1 ± 0.8	2.6 ± 0.5	2.4 ± 0.8	0.06
Bronchial bleeding	48 (64.8)	15 (55.5)	20 (86.9)	12 (50.0)	0.75
Mild	27 (36.4)	6 (22.2)	13 (56.5)	8 (33.3)	0.367
Moderate	21 (28.3)	9 (33.3)	7 (30.4)	4 (16.6)	0.186
Pneumothorax	5 (6.7)	0 (0)	0 (0)	5 (20.8)	**< 0.05**
Mild	3 (4.0)	0 (0)	0 (0)	3 (12.5)	**< 0.05**
Moderate	1 (1.3)	0 (0)	0 (0)	1 (4.1)	0.206
Severe	1 (1.3)	0 (0)	0 (0)	1 (4.1)	0.206

Highlighted in bold the areas where a p-value of less than 0.05 indicates a statistically significant difference

DL_CO_, diffusing capacity for carbon monoxide; FEV_1_, forced expiratory volume in 1 s; FVC, forced volume capacity; GGO, ground-glass opacities; HRCT, high-resolution computed tomography; KL-6, Krebs von den Lungen-6; LAS, large airway specimen; PFT, pulmonary function test; SP-D, surfactant protein D; UIP, usual interstitial pneumonia

### Diagnostic yield and histological diagnosis

A specific histological diagnosis was obtained in 47/74 cases (63.5%). The histological diagnostic yield tended to be statistically significantly higher in the LAS-negative group (40.7% in LAS positive group; 60.8% in LAS positive and negative group; 91.6% in LAS negative group) (Cochran-Armitage trend test). The histological interpretations are shown in Table 2 and include 11 (14.8%) UIP, 6 (8.1) probable UIP, 16 (21.6%) indeterminate for UIP, 9 (12.1%) non-specific interstitial pneumonia, 6 (8.1%) organizing pneumonia and 6 (8.1%) hypersensitivity pneumonitis. The institutional diagnosis after MDD is shown in Table 3 and include 9 (12.1%) idiopathic pulmonary fibrosis, 6 (8.1%) non-specific interstitial pneumonia, 4 (5.4%) organizing pneumonia, 24 (32.4%) unclassifiable and 9 (12.1%) hypersensitivity pneumonitis.

**Table 2:** Histological diagnosis

Histological diagnosis	No (%)
UIP	11 (14.8)
Probable UIP	6 (8.1)
Indeterminate for UIP	16 (21.6)
	– 14 NSIP/UIP
	– 1 NSIP/UIP/OP
	– 1 UIP/OP
Alternative diagnosis	
NSIP	9 (12.1)
OP	6 (8.1)
HP	6 (8.1)
Smoking-related IP	2 (2.7)
Others	7 (9.4)
	– 4 IgG4-related disease
	– 1 sarcoidosis
	– 1 cellular bronchiolitis
	– 1 malignancy
N.D.	11 (14.8)

DPB, diffuse panbronchiolitis; HP, hypersensitivity pneumonitis; N.D., non-diagnostic; NSIP, non-specific interstitial pneumonia; OP, organizing pneumonia; UIP, usual interstitial pneumonia

**Table 3:** MDD diagnosis

MDD diagnosis	No (%)
IPF	9 (12.1)
NSIP	6 (8.1)
OP	4 (5.4)
Unclassifiable	24 (32.4)
Hypersensitivity pneumonitis	9 (12.1)
CTD-ILD (MCTD/RA/SjS/SLE/SSc)	10 (13.5)
MPA	3 (4.0)
Others	9 (12.1)

CTD, connective tissue disease; IPF, idiopathic pulmonary fibrosis; MCTD, mixed connective tissue disease; MPA, microscopic polyangiitis; NSIP, non-specific interstitial pneumonia; OP, organizing pneumonia; RA, rheumatoid arthritis; SjS, Sjögren’s syndrome; SLE, systemic lupus erythematosus; SSc, systemic sclerosis

### Complications

In a comparison between the three groups, the LAS-negative group tended to have more pneumothorax complications than the other groups (0% in LAS positive group; 0% in LAS positive and negative group; 20.8% in LAS negative group) (Cochran-Armitage trend test). There was no clear difference in bronchial bleeding between the three groups (Table 1).

### Specimen characteristics

In total, 177 specimens were collected in the 74 cases, with a mean number of specimens per case of 2.3 ± 0.7 (Tables 1 and 4). 175 were collected with 1.9 mm probes and only 2 were collected with 2.4 mm probes. The most common biopsy lobe was the right lower lobe (63.8%), followed by the right upper lobe (15.2%) and the left lower lobe (14.1%). There were 85 (48.0%) LAS containing bronchial gland or bronchial cartilage: 30 containing both bronchial gland and bronchial cartilage, 50 containing only bronchial cartilage, and 5 containing only bronchial gland. The anatomical structures observed except for the large airway were alveoli (91.5%), bronchioles (49.1%), and perilobular area (43.5%), with few pleural specimens (1.2%). Ideal specimens were obtained in 47 of the 177 (26.5%) specimens, about a quarter of the total. A comparison of specimen characteristics showed that the proportion of bronchioles, alveoli, and perilobular area were significantly lower in the LAS-positive group than in the LAS-negative group (Table 3). Similarly, the ideal specimen ratio and the frequency of specimens of high pathological confidence were significantly lower in the LAS-positive group. The ratio of specimens with a pathological confidence level A was 25 of the 47 (53.1%) for ideal specimens and 43 of the 130 (33.0%) for unideal specimens (P < 0.05). In terms of the HRCT findings at the sample collection site, the LAS-positive group had a significantly higher proportion of traction bronchiectasis (65.5% vs. 29.6%) and diaphragm overlap sign (54.1% vs. 33.6%) than the LAS-negative group.

**Table 4:** Characteristics of specimens obtained by TBLC

Characteristics	All specimens	LAS positive	LAS negative	*P* value
Specimens, *n* (%)	177	85 (48.0)	92 (51.9)	
Maximum diameter, mm	6.4 ± 2.9	7.5 ± 3.7	5.3 ± 1.3	**< 0.05**
Biopsy lobe, *n* (%)				
Right upper lobe	27 (15.2)	7 (8.2)	20 (21.7)	**< 0.05**
Right middle lobe	6 (3.3)	2 (2.3)	4 (4.3)	0.684
Right lower lobe	113 (63.8)	58 (68.2)	55 (59.7)	0.275
Left upper lobe	6 (3.3)	2 (2.3)	4 (4.2)	0.684
Left lower lobe	25 (14,1)	16 (18.8)	9 (9.7)	0.129
Structures observed on the specimen				
Bronchiole	87 (49.1)	23 (27.0)	64 (69.5)	**< 0.05**
Alveoli	162 (91.5)	73 (85.8)	89 (96.7)	**< 0.05**
Perilobular area	77 (43.5)	15 (17.6)	62 (67.3)	**< 0.05**
Pleura	1 (1.2)	0 (0)	1 (1.0)	1
Ideal specimen, *n* (%)	47 (26.5)	5 (5.8)	42 (45.6)	**< 0.05**
Pathological confidence level				
Level A	68 (38.4)	23 (27.0)	45 (48.9)	**< 0.05**
Level B	84 (47.4)	48 (56.4)	36 (39.1)	**< 0.05**
Level C	10 (5.6)	5 (5.8)	5 (5.4)	1
Not evaluated	15 (8.4)	9 (10.5)	6 (6.5)	
HRCT findings in the biopsied lung segment, *n* (%)				
Honeycomb	8 (4.5)	4 (4.7)	4 (4.3)	1
Traction bronchiectasis	101 (57.0)	65 (76.4)	36 (39.1)	**< 0.05**
Reticulation	130 (73.4)	68 (80.0)	62 (67.3)	0.063
GGO	60 (33.8)	28 (32.9)	32 (34.7)	0.874
Consolidation	16 (9.0)	8 (9.4)	8 (8.6)	1
Nodule	14 (7.9)	4 (4.7)	10 (10.8)	0.167
Cyst	5 (2.8)	0 (0)	1 (1.0)	0.059
Diaphragm overlap sign, *n* (%)	77 (43.5)	46 (54.1)	31 (33.6)	**< 0.05**

Highlighted in bold the areas where a p-value of less than 0.05 indicates a statistically significant difference

GGO, ground-glass opacities; HRCT, high-resolution computed tomography; LAS, large airway specimen

## Discussion

To our knowledge, this is the first retrospective study of LAS obtained in TBLC, and it has revealed three important points. First, LAS are specimens that can be routinely collected by TBLC in patients with DPLD and most LAS are unideal specimens that do not contain bronchioles or perilobular area. Second, LAS are more likely to be collected in the presence of traction bronchiectasis or diaphragm overlap sign on HRCT of the biopsied lung segment. Third, TBLC cases that do not contain LAS tend to have a higher histological diagnostic yield.

Previous reports comparing the diagnostic yield of TBLC and SLB in DPLD suggested that specimens containing large airway or pleura, specimens with non-specific pathological findings or hemorrhage artifacts, and normal lung tissue were recognized as unideal specimens that may not contribute to the histological diagnosis [6, 7, 9, 18]. However, in terms of the definition of an ideal specimen, most of the studies defined adequacy as the inclusion of alveoli in the specimen and did not define whether there were concomitant bronchioles, perilobular area, or pleura [13]. However, pathological evaluation of specimens largely occupied by alveoli may be unideal for some histological patterns in which lesions are located also in the bronchiole or perilobular area (e.g., UIP pattern, UIP/NSIP pattern, hypersensitivity pneumonitis, sarcoidosis). Therefore, we considered that the conventional definition of only the presence of alveoli as a requirement for an ideal specimen is insufficient and inaccurate. And we investigated the possibility that TBLC specimens containing all of bronchioles, alveoli, and perilobular area would be ideal for histological diagnosis. Based on this definition, we attempted to clarify the exact role of LAS in the present TBLC study.

To date, only two reports have referred to specimens from the large airway in TBLC. Ussavarungsi et al. [10] reported that specimens including large airway were found in 47% of TBLC cases, and Pajares et al. reported that specimens including bronchial wall were found in 8.6% of cases [11]. However, the definition of LAS was not rigorous, and we defined LAS as specimens that include bronchial gland or bronchial cartilage based on the results of a previous anatomical study [15]. In the present study, LAS were found in 67.5% of TBLC cases and 48.0% of TBLC specimens under the usual TBLC procedure, indicating that LAS are not infrequent and can be commonly collected in TBLC. Besides, most of the LAS were unideal specimens (94.2%), and the difference in the proportion of ideal specimens in the LAS-negative group was statistically significant (Table 4). We must also mention that there is uncertainty about whether the method of specimen evaluation described above is universal because no similar studies have been conducted previously, to our knowledge. However, we believe that these definitions will be helpful in creating a smoother and less misunderstood discussion of diagnostic yield and the adequacy of TBLC specimens.

We reviewed the HRCT findings in the biopsied lung segment where LAS were collected and obtained two interesting findings. First, the rate of LAS collection increased when traction bronchiectasis was present (Table 4). In addition to previous reports cautioning that dense honeycomb lesions or dense fibrotic lung parenchyma lesions should be avoided in TBLC sampling [12, 19], we speculate that optimal sampling sites in TBLC may be in areas mainly ground-glass opacities and reticular shadows where architectural distortion such as traction bronchiectasis or honeycomb is not evident [10]. Second, the presence of a diaphragm overlap sign increases the ratio of LAS collection. HRCT, which is usually performed before TBLC, is performed on deep inspiration with the patient in the awake state. However, during the actual TBLC procedure, deep sedation is often used to reduce pain, and patients rarely inhale deeply or stop breathing. In other words, it is common for TBLC to be performed in situations where the precise location of the biopsy is more difficult to determine than when HRCT is performed. Therefore, we believe that if the area to be sampled at the time of HRCT overlaps the sub-diaphragm area, there is a greater possibility that the area will be hidden by the diaphragm at the time of TBLC. If the biopsy site overlaps the sub-diaphragmatic area, determination of whether the cryobiopsy probe has reached just below the pleura can be inaccurate, leading to an inaccurate biopsy of the peripheral lung. To predict whether such a precarious situation would occur, we established the diaphragm overlap sign and found that the percentage of LAS collected increased when the sign was positive. Although the fluoroscopic guidance is widely used in bronchoscopy, its accuracy is limited to the lateral lung area [20]. The use of cone beam CT-guided TBLC (CBCT-guided TBLC) has recently been reported to reduce complications such as pneumothorax and hemorrhage and improve diagnostic accuracy because the distance between the probe and pleura can be more accurately determined [21–23]. CBCT-guided TBLC is not a universal technique because it is performed under general anesthesia in a hybrid operating room. However, we believe that these reports are significant in that they demonstrate the usefulness of multi-dimensional (e.g.; axial, coronal, sagittal) guidance. Therefore, for fluoroscopic TBLC performed under the guidance of coronal view only, it is advisable to confirm the expected position of the pleura-probe in the axial and sagittal images of HRCT in advance, and to avoid biopsy of the area with positive diaphragm overlap sign.

Although the impact of the presence of LAS on the histological diagnostic yield has not been studied before, our study suggested that LAS had apparent effect on the rate of histological diagnosis (Table 1). On the other hand, about half of the biopsy specimens were LAS-positive, and we believe that the optimal number of specimens should be investigated. The average number of specimens collected was 2.7 in this study, and we collected a total of 2 or more specimens from different lung segments in 66/74 (89.1%) cases. 23 of the 51 LAS-positive cases had both LAS-positive and LAS-negative specimens in the same TBLC procedure. Therefore, it is important for pulmonologists to recognize that there is always the possibility of LAS in individual biopsy specimens and to perform TBLC so that multiple biopsies do not diminish the accuracy of histological diagnosis. Previous reports by Ravaglia et al. that obtaining two specimens from different segments within the same lobe may reduce sampling error and increase diagnostic yield would also support our current considerations [17, 24].

This study has several limitations. First, this is a retrospective observational study with a small number of cases from a single center. Second, the pathological evaluation was performed by a single pathologist. Considering the low rate of concordance between pathologists in the histological evaluation of TBLC specimens, it would be necessary for multiple pathologists to perform the histological evaluation. Third, several new pathological definitions (adequacy of a specimen and LAS) and new HRCT definition (diaphragm overlap sign) were used in the present study, but these are not generalized concepts or indicators, and their validity needs to be evaluated by pathologists and radiologists in the future. Finally, we did not compare the pathological findings of SLB in the same case. This study was conducted from the viewpoint of LAS as one of the factors involved in the diagnostic discrepancy between SLB and TBLC, and a comparative study of the characteristics of specimens and LAS collection ratios of both SLB and TBLC will further clarify the reasons behind the difference in diagnostic accuracy.

## Conclusion

In conclusion, this study showed that LAS obtained in TBLC have a high frequency of unideal specimens that do not include the bronchiole or perilobular area. Since the histological diagnostic yield tends to be higher in cases that do not contain LAS, it may be important to determine the biopsy site that reduces the frequency of LAS collection by referring to the HRCT findings in TBLC.

## Data Availability

The dataset supporting the conclusions of this article is included within the article.

## Abbreviations

DPLD: Diffuse parenchymal lung disease
HRCT: High-resolution computed tomography
LAS: Large airway specimen
SLB: Surgical lung biopsy
TBLC: Transbronchial lung cryobiopsy
UIP: Usual interstitial pneumonia
